# Interventions for prudent antibiotic use in primary healthcare: an econometric analysis

**DOI:** 10.1186/s12913-020-05732-2

**Published:** 2020-09-23

**Authors:** Elina Lampi, Fredrik Carlsson, Pär-Daniel Sundvall, Marcela Jaime Torres, Peter Ulleryd, Christina Åhrén, Gunnar Jacobsson

**Affiliations:** 1 Department of Economics, University of Gothenburg, Vasagatan 1, SE-411 24 Gothenburg, Sweden; 2 Center for Antibiotic Resistance Research (CARe), University of Gothenburg, Guldhedsgatan 10, 405 30 Gothenburg, SE Sweden; 3 Region Västra Götaland, Research and Development Primary Health Care, Research and Development Centre Södra Älvsborg, Sven Eriksonsplatsen 4, SE-503 38 Borås, Sweden; 4 Department of Public Health and Community Medicine/Primary Health Care, Institute of Medicine, Sahlgrenska Academy, University of Gothenburg, Box 454, SE-405 30 Gothenburg, Sweden; 5 School of Management and Business, Research Nucleus on Environmental and Natural Resource Economics (NENRE), Universidad de Concepción, Victoria 471, Barrio Universitario, Concepción, Chile; 6 Department of Communicable Disease Control and Prevention, Region Västra Götaland, Kaserntorget 11B, SE-411 18 Gothenburg, Sweden; 7 Swedish Strategic Program against Antimicrobial Resistance (Strama), Region Västra Götaland, Bergslagsgatan 2, SE-411 04 Gothenburg, Sweden; 8 Department of Infectious Diseases, Institute of Biomedicine, Sahlgrenska Academy, University of Gothenburg, Medicinaregatan 9 A-B, SE-413 90 Gothenburg, Sweden; 9 Department of Infectious Diseases, Skaraborg Hospital, Lövängsvägen, SE-541 42 Skövde, Sweden

**Keywords:** Health services research, Primary healthcare, Quality improvement, Antibiotics, Intervention, Self-evaluation

## Abstract

**Background:**

Rational antibiotic prescribing is crucial to combat antibiotic resistance. Optimal strategies to improve antibiotic use are not known. Strama, the Swedish strategic program against antibiotic resistance, has been successful in reducing antibiotic prescription rates. This study investigates whether two specific interventions directed toward healthcare centers, an informational visit and a self-evaluation meeting, played a role in observed reduction in rates of antibiotic prescriptions in primary healthcare.

**Methods:**

The study was a retrospective, observational, empirical analysis exploiting the variation in the timing of the interventions and considering past prescriptions through use of estimations from dynamic panel data models. Primary healthcare data from 2011 to 2014 were examined. Data were from public and private primary healthcare centers in western Sweden. The key variables were prescription of antibiotics and indicator variables for the two interventions.

**Results:**

The first intervention, an educational information intervention, decreased the number of prescriptions among public healthcare centers, but this effect was only temporary. We found no proof that the second intervention, a self-evaluation meeting at the healthcare center, had an impact on the reduction of prescriptions.

**Conclusions:**

Single educational interventions aimed at influencing rates of antibiotic prescriptions have limited impact. A multifaceted approach is needed in efforts to reduce the use of antibiotics in primary health care.

## Background

Antibiotic resistance is a global problem with important medical and economic implications. The more antibiotics used, the more likely development of resistant microorganisms [1–5]. Antibiotics and other medicines are often prescribed too easily for a number of reasons, including the desire to have a good relationship with patients [6, 7], the belief that patients expect to receive medications [8–12], time pressure and inadequate policies or guidelines [13] ignorance among practitioners regarding the conditions leading to resistance [12] and the way the healthcare system works. It may be less convenient for the physician to wait and see how an illness develops and whether there is need for antibiotics than to simply write a prescription right away [13].

Conservation, decreasing the use of antibiotics through different means, slows down the development of resistance and provides more time to develop alternatives [14]. Even if antibiotic resistance is a global problem, its solutions happen at the national and regional levels [15]. Education of healthcare providers, and the public on correct antibiotic use and problems with antibiotic resistance is a central form of conservation [16]. Educational strategies include meetings with didactic lectures and distribution of informational leaflets. A Cochrane review [17] reports improved antibiotic use in five of six studies with dissemination of educational materials in printed form or meetings; the median effect size based on the type of study ranged from about 11 to 43%. However, the effect of educational activities is often found to be transient [18] and should be combined with active interventions. Use of rapid diagnostic tests such as the concentration of C-reactive protein as a point-of-care test, and rapid antigen detection test for group A streptococci [19], and academic detailing, or face-to-face education [20], have been found to have a positive effect on appropriate prescribing of antibiotics compared with personnel meetings and educational materials.

The level of antibiotic prescription is low in Sweden compared with many other European countries [21]. However, there are differences in rates of prescriptions between different regions and municipalities. Hedin et al. [22] compared municipalities in Sweden with high and low antibiotic prescription rates and found that neither socioeconomic factors nor differences in infection symptoms or number of physician consultations could explain these differences. They concluded that reasons for these prescription rate differences are unclear, but they might be due to differences in prescribing behavior of physicians.

The Strama Västra Götaland, the Swedish strategic program against antibiotic resistance, performed two different interventions, among other initiatives, targeting physicians and nurses in nearly all primary health care centers (PHCCs) in Region Västra Götaland in western Sweden during 2012–2014. Intervention A consisted of a visit from a peer at each center informing about rational use of antibiotics and how the center prescribed compared with others. Intervention B was initiated by Strama but conducted by the center itself; physicians shared their prescribing rates and discussed how well they had succeeded in following the guidelines for antibiotic prescription.

It was not clear from previous studies which one should have had more impact. For example, Laxminarayan et al. [16] argue that physicians are influenced by their peers, whereas Persell et al. [23] find no effect of peer comparisons on the level of antibiotic prescription. However, according to Elster [24] appealing to norm-based behavior could produce moral costs or benefits (i.e., feelings of guilt or self-respect) when individuals fail or succeed to conform to the expected behavior. If so, we hypothesized that intervention A could have a larger impact on prescribing behavior than intervention B because intervention A appeals to norm-based behavior. By being exposed to intervention A, personnel at the PHCC became aware that their prescribing behavior had been observed. In contrast, intervention B did not provide the personnel with such scrutiny.

In this study, we investigate whether the two interventions affected the prescription rate at PHCC and, if so, whether there were differences in terms of effects of the two interventions between public and private healthcare centers. We investigate both prescriptions of all antibiotics and of antibiotics related to respiratory tract infections. Our empirical strategy exploits the variation in the timing of the interventions.

## Methods

### Description of the two interventions

In the second quarter of 2012, Strama Västra Götaland initiated two interventions directed toward PHCCs in the Västra Götaland region. In intervention A, a Strama peer visited each healthcare center and informed physicians and nurses about appropriate antibiotic prescribing behavior and how the center prescribed compared with other PHCCs in the region. Intervention B was initiated by Strama but conducted by the healthcare centers themselves, where clinicians shared their prescribing rates for different indications and antibiotics. They also discussed how well they had succeeded in following the guidelines for antibiotic prescription and possible ways to improve. Each PHCC was asked to send a report on this self-evaluation to Strama. After doing so, the center received a reward of a fixed sum plus an adjusted amount based on the number of listed patients at the healthcare center.

The initial selection of PHCCs that would get a visit by Strama was not random; instead, centers that expressed an interest and those with high prescription levels were more likely to be targeted first. Selection later was also not randomized. At the end of 2012, about 35% of the centers had been visited. At the beginning of 2013, two things happened: more PHCCs were targeted by intervention A, and intervention B was started. As of the second quarter of 2014, 97 and 92% of the PHCCs had been targeted by interventions A and B, respectively. The interventions where never done simultaneously. However, they could have happened during the same observed quarter.

### Description of the sample

We have data on prescription of all types of antibiotics and those related to treatment of respiratory tract infection at 206 PHCCs between the first quarter of 2011 and the second quarter of 2014. From the second quarter of 2011 and onwards, we also have information about characteristics of the PHCCs The main analysis is based on the balanced panel-that is, we included only healthcare centers for which we have observations for the whole-time period from the third quarter of 2011 (because of the need to include a lagged dependent variable in the analysis) to the fourth quarter of 2013.

### Characteristics of PHCC

We investigated whether there were statistically significant differences between characteristics between private and public PHCC and between the last quarters of 2011 and 2013, using a t-test for continuous variables and Wilcoxon rank-sum test for discrete variables (shares).

### Econometric framework

Using an econometric analysis, we investigated the effects of the two interventions on prescriptions while controlling for the relationship between characteristics of the healthcare centers and prescription levels, in general, and the difference between private and public PHCCs.

We began by investigating the effects of the two interventions on total antibiotics prescriptions per patient visits. In addition, we investigated the level of respiratory tract infection related antibiotic prescriptions per patient visits of an average PHCC in Region Västra Götaland. We focused on respiratory tract infection related antibiotic prescriptions per patient visits because there is evidence that the rate of inappropriate prescriptions is higher in respiratory tract infections than in other type of infections [25]. We used respiratory tract infection related antibiotics as a proxy for respiratory tract infections, we are aware that these antibiotics to a lesser extent are used in other infections as well.

Because the assignment of PHCCs in both interventions was to some extent based on their past levels of prescriptions, and given the panel structure of the data, we evaluated the effects of the interventions by means of linear dynamic panel-data models. These models include p lags of the dependent variable as covariates and contain unobserved panel-level effects to take account of the feedback from past prescriptions. The specification to be estimated is presented as follows:

1 $$ {\displaystyle \begin{array}{c}{y}_{it}={\rho y}_{i,t-1}+{\alpha}_1{T}_{1i}+{\alpha}_2{T}_{2i}+\beta {x}_{it}+{\mu}_t+{v}_i+{\varepsilon}_{it}\\ {}\left(i=1,\dots, N;t=1,\dots, T\right),\end{array}} $$

where *y*_*it*_ denotes PHCC, *i*’s total antibiotics prescriptions per patient visits/respiratory infection related antibiotic prescriptions per 100 visits^1^ in quarter *t*, *y*_*i,t–*1_ is the level of prescriptions of healthcare center *i* in quarter *t*–1; *T*_1*i*_ and *T*_2*i*_ are intervention status indicators that are equal to 1 if the healthcare center was targeted by intervention A or B, respectively, and 0 otherwise; and *x*_*it*_ is a vector of characteristics at both the center and patient levels, which are intended to capture the observed time-varying selection criteria used by Strama to determine the treatment status of clinics. Similarly, μ_t_ denotes quarterly dummy variables accounting for seasonal effects of antibiotic prescriptions; *v*_*i*_ are panel-level unobserved effects that are correlated with the lagged dependent variable and take into account unobserved characteristics of healthcare centers, such as the motivation of directors to participate in the interventions; and *ε*_*it*_ is the error term. The direct effects of the interventions are estimated by the parameters α_1_ and α_2_. Standard errors include the Windmeijer’s finite-sample correction, which are suitable in presence of both heteroskedasticity and autocorrelation [26].

This equation is estimated by means of the extended system generalized method of moments estimator (GMM) derived by Arellano and Bover [27]) and Blundell and Bond [28]), an estimator that is suitable with data with few time periods and many panels. It uses extra moment conditions and lagged differences of *y*_*it*_ as instruments for equations in levels, as well as lagged levels of *y*_*it*_ as instruments for equations in first differences. The use of the system GMM estimator has several advantages with respect to the standard first differences GMM, including substantial efficiency gains, which makes the level restrictions informative even in the presence of weak instruments [29].^2^

We estimated eq. (1) for all, public, and private centers. Moreover, we evaluated the duration of the effects by separating the total effects of the interventions into short-run effects (i.e., separate effects for the intervention quarter and the first quarter after the intervention) and long-run effects (the remaining quarters).

We then investigated the effects of the two interventions on PHCCs with similar characteristics. We were interested mainly in public and private centers, but also those that did not already have low levels of antibiotic prescription before the interventions started. We therefore also analyzed the prescribing behavior of centers when removing the centers with low levels of prescription, in our case the bottom quartile with the lowest levels. This gave us information on the effects of the interventions when the type of the PHCC matters.

### Definition of respiratory tract infection related antibiotics according to anatomical therapeutic chemical classification (ATC)

Phenoxymethylpenicillin (ATC J01CE02), doxycycline (ATC J01CE02), amoxicillin (ATC J01CA04), cephalosporines (J01DB-DE), macrolides (ATC J01FA), amoxicillin with beta-lactamase inhibitor (ATC J01CR02).

## Results

Table 1 reports descriptive statistics for all the PHCCs, as well as for public and private centers separately. Private healthcare centers were on average smaller, measured as number of patient visits, but they had a higher number of antibiotic prescriptions per 100 visits. However, private centers also had a slightly but statistically significantly higher share of patients with visits due to an infection. Still, the level of respiratory tract infection related antibiotic prescriptions per 100 infections was larger at private PHCCs. For patient composition, in terms of socioeconomic background, we could not find any statistically significant differences considering the care need index (CNI) between public and private centers. On the other hand, public centers had more patients with comorbidities (adjusted clinical groups).

**Table 1:** Characteristics of healthcare centers

Variable	All centers		Public centers		Private centers		Pub. vs. priv. 2011	Pub. vs priv. 2013
	2011	2013	2011	2013	2011	2013	*p*-value	*p*-value
Private healthcare center^a^	0.41 (0.49)	0.40 (0.49)						
No. of visits at center	5501 (2708)	5578 (2481)	6354 (2596)	6263 (2362)	4260 (2380)	4553 (2308)	<0.001	<0.001
No. of listed patients	8215 (3908)	8239 (3725)	9363 (3883)	9173 (3704)	6546 (3316)	7070 (3408)	<0.001	<0.001
No. of antibiotic prescriptions per 100 visits	9.55 (3.46)	6.54 (1.67)	8.25 (1.78)	6.12 (1.32)	11.44 (4.34)	7.17 (1.94)	<0.001	0.001
No. of respiratory tract infection related antibiotics prescriptions per 100 visits	5.76 (2.76)	3.17 (1.11)	4.68 (1.30)	2.88 (0.82)	7.34 (3.48)	3.61 (1.33)	<0.001	<0.001
No. of antibiotic prescriptions per 100 listed patients	6.39 (2.37)	4.41 (1.22)	5.72 (1.47)	4.31 (1.16)	7.39 (3.01)	4.57 (1.28)	<0.001	0.048
No. of respiratory tract infection related antibiotics prescriptions per 100 listed patients	3.85 (1.86)	2.13 (0.73)	3.23 (0.97)	2.02 (0.64)	4.75 (2.42)	2.29 (0.83)	<0.001	0.018
Share infection visits as percentage of total visits	0.24 (0.05)	0.19 (0.05)	0.22 (0.04)	0.18 (0.04)	0.26 (0.06)	0.20 (0.05)	<0.001	0.001
Care need index (CNI)^b^	2.35 (0.72)	2.30 (0.77)	2.37 (0.65)	2.30 (0.68)	2.33 (0.81)	2.29 (0.89)	0.923	0.503
Adjusted clinical groups^c^	1.01 (0.14)	1.01 (0.13)	1.02 (0.13)	1.03 (0.01)	0.98 (0.17)	0.98 (0.14)	0.063	0.184
Share younger patients (<19 years) as percentage	13.1 (3.89)	13.4 (3.79)	14.1 (2.80)	14.2 (2.87)	11.7 (4.74)	12.0 (4.55)	<0.001	<0.001

*Note*: Mean values and standard deviations (in parentheses) are presented for the entire sample of all healthcare centers and for public and private healthcare centers separately

^a^ = 1 if private healthcare center

^b^Index to capture how socioeconomic status of listed patients, such as unemployed, single parent, single person over 65 years old, low education level, and children <5 years old, affect health (National Board of Health and Welfare 2004). A higher value indicates worse socioeconomic factors

^c^Index for health status of listed patients, grouping patients based on all their diagnoses, the seriousness and longevity of the diseases, and their need for healthcare resources. Those who have only one diagnosis belong to one group, those with two diagnoses are in another group, those with three to four diagnoses are in a third group, and so on (National Board of Health and Welfare 2004). A higher value indicates a greater need for healthcare resources

The number of antibiotic prescriptions had decreased almost 32% for the whole sample, i.e. from an average of 9.55 in 2011 to 6.54 in 2013 per 100 visits (of any type). The decrease was larger at private (37%) than at public (26%) healthcare centers, which means that the difference in prescriptions between public and private healthcare centers was considerably smaller in 2013 than in 2011. If we instead focus on respiratory tract infection related prescriptions, the number of prescriptions has decreased from an average of 5.76 in 2011 to 3.17 in 2013 per 100 visits, a 45% decrease. Thus, the decrease in respiratory tract infection related prescriptions was larger than the overall decrease in prescriptions. Again, the decrease was larger at private (51%) than at public (28%) healthcare centers.

**Fig. 1:**
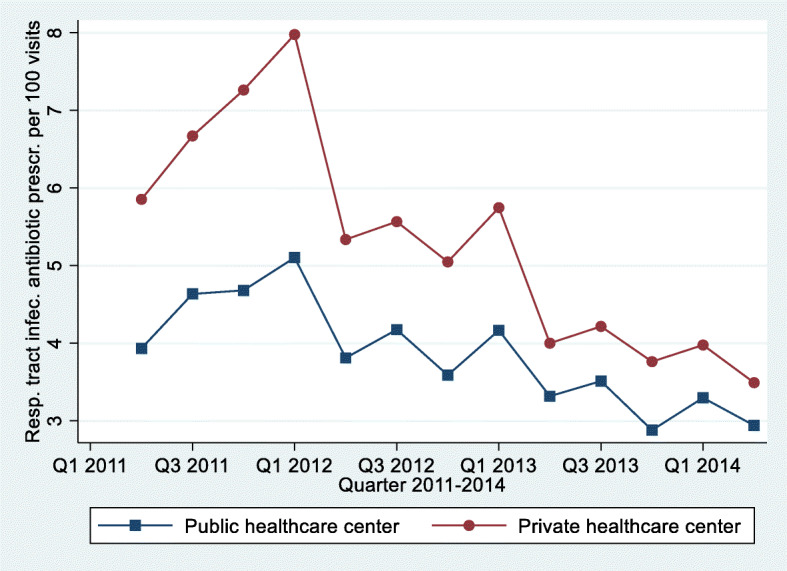
Number of respiratory tract infection related antibiotic prescriptions per 100 patient visits for each quarter

The average number of respiratory tract infection related antibiotic prescriptions per 100 infection visits and quarter for both types of centers is plotted in Fig. 1.

We can see two important things from the graph: private centers had higher levels of antibiotic prescriptions than public centers, but the difference was decreasing over time, and there was an overall downward trend in the prescription of respiratory tract infection related antibiotics. A similar pattern was observed for all antibiotics per patient visits at these centers.

### Impacts of the two interventions

Results are presented in Table 2. We first report results for prescriptions of all kinds of antibiotics per 100 visits. Then, since the rate of inappropriate prescriptions is suspected to be higher in respiratory tract infections than in other type of infections [25] we also report results for respiratory tract infection related antibiotics prescriptions per 100 visits. Moreover, we also estimated both types of antibiotics prescriptions per number of listed patients instead of per patient visits (see Table A1 in the Appendix). The columns (1) and (2) of the table correspond to the whole sample (i.e., all centers) when the characteristics of PHCCs are excluded and included as covariates, respectively. Columns (3) and (4) correspond to the subsamples of public and private healthcare centers, respectively.

**Table 2:** Effects of interventions on antibiotics prescription per 100 patient visits

	Total prescriptions				Respiratory tract infection related prescriptions			
	Whole sample	Whole sample	Public centers	Private centers	Whole sample	Whole sample	Public center	Private center
Prescription previous	0.615^***^	0.605^***^	0.265^***^	0.674^***^	0.650^***^	0.631^***^	0.279^***^	0.690^***^
quarter	(0.0645)	(0.0702)	(0.0497)	(0.0669)	(0.0515)	(0.0648)	(0.0577)	(0.0607)
Intervention A (same	−0.145	− 0.124	− 0.312^*^	− 0.0689	− 0.100	− 0.0782	− 0.189	0.00285
quarter)	(0.168)	(0.179)	(0.144)	(0.313)	(0.124)	(0.131)	(0.104)	(0.232)
Intervention A (after one	−0.0956	− 0.0359	− 0.401^*^	0.0101	− 0.270^*^	− 0.188	−0.421^***^	− 0.0891
quarter)	(0.200)	(0.223)	(0.190)	(0.392)	(0.133)	(0.147)	(0.121)	(0.268)
Intervention A (remaining	0.213	0.376	−0.120	0.464	0.0182	0.247	−0.184	0.454
quarters)	(0.213)	(0.251)	(0.223)	(0.510)	(0.151)	(0.182)	(0.136)	(0.403)
Intervention B (same	0.140	0.120	−0.00626	0.160	0.223	0.187	0.0238	0.313
quarter)	(0.260)	(0.259)	(0.234)	(0.441)	(0.214)	(0.210)	(0.149)	(0.397)
Intervention B (after one	0.564	0.509	0.268	0.477	0.599	0.529	0.263	0.701
quarter)	(0.407)	(0.403)	(0.401)	(0.707)	(0.333)	(0.324)	(0.243)	(0.663)
Intervention B (remaining	0.972	0.875	0.767	0.412	0.702	0.638	0.583	0.421
quarters)	(0.560)	(0.557)	(0.597)	(0.832)	(0.415)	(0.407)	(0.351)	(0.753)
Low care need index (CNI)		0.311	−0.566	0.820		0.295	−0.417	0.684
		(0.460)	(0.313)	(0.717)		(0.331)	(0.290)	(0.504)
High care need index (CNI)		0.589	0.475	0.670		0.239	0.270	0.162
		(0.342)	(0.441)	(0.477)		(0.283)	(0.322)	(0.426)
Adjusted clinical groups		−1.683	−2.982^*^	−1.477		−3.200^*^	−1.941	−3.974
		(2.010)	(1.164)	(2.735)		(1.586)	(1.069)	(2.084)
Share younger patients		0.208	−0.150	0.377		0.249^*^	−0.0358	0.326*
		(0.146)	(0.116)	(0.197)		(0.108)	(0.0818)	(0.145)
No. of listed patients		−0.144	−0.227^*^	− 0.0384		− 0.0777	− 0.0440	0.0172
		(0.107)	(0.106)	(0.165)		(0.106)	(0.0858)	(0.140)
Constant	4.992^***^	4.920	14.25^***^	1.953	2.492^***^	3.020	6.441^***^	2.496
	(0.503)	(3.447)	(2.276)	(4.259)	(0.233)	(2.238)	(1.725)	(2.921)
Quarterly dummies	Yes	Yes	Yes	Yes	Yes	Yes	Yes	Yes
Observations	1888	1888	1120	768	1888	1888	1120	768
Number of centers	189	189	112	77	189	189	112	77

Robust standard errors in parentheses

*** *p* < 0.001, ** *p* < 0.01, * *p* < 0.05

First, the model results show that there is a positive and statistically significant effect of the previous quarter’s level of prescriptions on the current quarter’s prescription level both for total prescriptions and for respiratory tract related antibiotics. This result holds even after controlling for characteristics of the health centers, and the effect is clearly stronger, twice as large, among private healthcare centers than among public ones.

Our main interest is on the potential effects of the two interventions. We first focus on the results for the whole sample and total antibiotics prescriptions. None of the six intervention coefficients are statistically significant in the first or the second model. Among the healthcare center characteristics, the coefficients for the adjusted clinical groups and the size of the PHCC are statistically significant and negative. This indicates that a center with more patients with comorbidities has a lower level of antibiotic prescriptions and that centers with a relatively smaller number of patients have higher level of prescriptions. The quarter-by-year dummies are all negative and highly statistically significant. Since the reference quarter is the third quarter of 2011, the negative signs reflect the downward trend of prescriptions that we saw when looking at the raw data. For the general model in the whole sample, we can thus draw the conclusion that the interventions do not seem to have any statistically significant effect on antibiotics prescriptions in general.

As shown in Table 1, private healthcare centers are on average smaller, have a different patient composition, and prescribe more antibiotics compared with public centers. We therefore estimated separate models for public and private healthcare centers in columns (3) and (4). Here we did find a statistically significant effect of the first intervention for the public healthcare centers. During the first quarter after intervention A took place, the number of antibiotic prescriptions was lower at these centers. The effect size was not large, prescriptions at public centers with the intervention were on average 5% lower during the first quarter after the intervention (using a mean value of prescriptions for the whole timer period of 7.55 prescriptions per 100 patient visits). Note that this decrease is additional to the downward trend of prescriptions that had already started before the interventions took place, since the model controls for both the time trend and differences in healthcare center characteristics. For the second intervention, we did not find any statistically significant effect. For private centers, we do not find any statistically significant effects for either of the two interventions. Although prescriptions have gone down more at private centers, this cannot be directly attributed to either of these specific interventions.

Next, we present the results for antibiotics prescribed for respiratory tract related infections. For the whole sample we observe a statistically significant short-term decrease in prescriptions during the first quarter after intervention A, but the effect disappears when we add controls for PHCC characteristics. Again, the comorbidities are significantly and negatively correlated with the level of prescriptions. Finally, we confirm the previous results for interventions for public and private health care centers: Intervention A had a short-lasting negative effect on prescription rates in public centers while we did not find support for an effect of intervention B. The effect size is larger for respiratory tract related infections: prescriptions at public centers with the intervention were on average 10.5% lower during the first quarter after the intervention (using a mean value of prescriptions for the whole timer period of 3.98 prescriptions per 100 patient visits). Our results persist regardless if we look at antibiotics prescriptions in general or prescriptions targeted to only respiratory tract infections. The results are not either sensitive if we estimate antibiotics prescriptions per visit or per number of listed patients (see Table A1 in the Appendix).^3^

### Sensitivity analysis

Since the level of prescriptions in the previous quarter is an important factor for prescription in the current quarter, as shown for all models in Table 2, it is also of interest to investigate whether the effect of the intervention is stronger if we remove healthcare centers that already had a low level of prescriptions before the interventions took place. In addition, the average number of prescriptions is considerably higher at the private centers. However, given the small number of observations and the short time period during which intervention B was implemented, we cannot make a disaggregate analysis. What we do as a sensitivity analysis is to classify healthcare centers based on the level of prescriptions before the interventions (i.e., in 2011). In order to be able to compare we classify centers with total antibiotic prescriptions at the bottom quartile are defined as low prescription centers; the average number of total antibiotics prescriptions per 100 patient visits is lower than 7.4. Results after the centers with the lowest levels of prescriptions are removed are presented in Table 3.

**Table 3:** Effects of interventions on prescriptions/100 patient visits without centers with lowest levels of prescriptions

	Total prescriptions			Respiratory tract infection related prescriptions		
	Whole sample	Public centers	Private centers	Whole sample	Public centers	Private centers
Prescription previous quarter	0.594***	0.204***	0.662***	0.636***	0.227***	0.694***
	(0.0805)	(0.0541)	(0.0725)	(0.0715)	(0.0623)	(0.0627)
Intervention A (same quarter)	− 0.124	−0.440***	0.0602	− 0.0350	− 0.185	0.120
	(0.216)	(0.163)	(0.351)	(0.158)	(0.129)	(0.260)
Intervention A (after one quarter)	−0.0911	−0.601***	0.0989	−0.163	− 0.467***	0.0637
	(0.275)	(0.221)	(0.465)	(0.178)	(0.144)	(0.315)
Intervention A (remaining quarters)	0.286	−0.247	0.544	0.273	−0.203	0.687
	(0.313)	(0.271)	(0.613)	(0.234)	(0.166)	(0.485)
Intervention B (same quarter)	−0.0946	− 0.0924	− 0.123	− 0.00975	− 0.0262	0.0662
	(0.306)	(0.292)	(0.427)	(0.254)	(0.183)	(0.381)
Intervention B (after one quarter)	0.293	0.114	0.141	0.351	0.254	0.398
	(0.484)	(0.506)	(0.671)	(0.398)	(0.308)	(0.628)
Intervention B (remaining quarters)	0.536	0.517	−0.117	0.302	0.525	−0.0316
	(0.675)	(0.761)	(0.794)	(0.503)	(0.442)	(0.701)
Low care need index (CNI)	0.636	−0.136	1.059	0.600*	−0.0329	0.910*
	(0.488)	(0.309)	(0.756)	(0.361)	(0.280)	(0.537)
High care need index (CNI)	0.372	0.00991	0.407	−0.169	−0.764	− 0.0491
	(0.456)	(0.747)	(0.536)	(0.374)	(0.525)	(0.453)
Adjusted clinical groups	−0.142	−3.723**	0.843	−2.199	−1.318	−2.999
	(2.431)	(1.635)	(3.088)	(1.961)	(1.359)	(2.433)
Share younger patients	0.277*	−0.227	0.416**	0.304**	−0.0299	0.361**
	(0.166)	(0.166)	(0.196)	(0.131)	(0.105)	(0.152)
No. of listed patients	−0.131	−0.276**	−0.00495	− 0.0464	−0.0355	0.0620
	(0.130)	(0.132)	(0.171)	(0.145)	(0.111)	(0.161)
Constant	2.926	17.67***	−0.747	1.386	6.569***	0.893
	(4.177)	(3.599)	(4.800)	(2.780)	(2.304)	(3.277)
Quarterly dummies	Yes	Yes	Yes	Yes	Yes	Yes
Observations	1429	730	699	1429	730	699
Number of id	143	73	70	143	73	70

Robust standard errors in parentheses

*** p < 0.01, ** p < 0.05, * *p* < 0.1

The results of Table 3 confirmed the results of Table 2. There was again little support for any effects of intervention A, and no support for the intervention B. What we find is again an initial negative short-term effect of intervention A on public PHCCs: The number of prescriptions of all kinds of antibiotics declined temporarily during the first six months and the respiratory tract related antibiotics declined during the first three months after the intervention A. On the other hand, the results in Table 3 also show a significant increase in the prescription rate in the respiratory tract related antibiotics about a half year after the intervention A, a result that is primarily caused by an increase among the private centers. Both public and private centers with patients with more comorbidities prescribe less, while private centers with large share of young patients prescribe more.

## Discussion

Our study shows that an intervention with an educational information at the PHCC, decreased the number of prescriptions among public healthcare centers, but this effect was only temporary. We found no evidence that the intervention with a self-evaluation meeting at the healthcare center, had an impact on the reduction of prescriptions. The number of respiratory tract infection related antibiotic prescriptions per 100 visits has decreased by almost 45% between 2011 and 2013 in the Region Västra Götaland in western Sweden. This must be viewed as a successful antibiotic stewardship initiative. The number of total antibiotic prescriptions per 100 visits decreased with 32%. Despite the substantial reduction in total antibiotic prescriptions and respiratory tract infection antibiotics, where the rate of inappropriate prescriptions is suspected to be higher than in other type of infections, we find little support that either of the specific targeted interventions by themselves, an informational visit and a self-evaluation meeting, played a long-lasting role in this downward trend in either public or private health centers. In public centers, an intervention with an informational visit had a transient impact: the number of prescriptions, especially the number of respiratory tract infection related antibiotic prescriptions, was lower after a Strama peer had visited a PHCC, a result that holds even when controlling for the downward time trend in prescriptions and for various healthcare center characteristics. The effect was only temporary and disappeared after about half a year, confirming the previous findings by Dellit et al. [18] that the effect of educational activities is often transient. Notwithstanding the short-lived effect, this result is in line with our predictions, suggesting that changing norms could play a crucial role in keeping antibiotic prescription efficient. As pointed out earlier, we expected that because intervention A appeals to norm-based behavior, it would be more likely to affect prescribing behavior than intervention B. However, because it involved only one visit from Strama, personnel actions were not scrutinized for a longer time, reducing the incentives to change behavior further. This could explain why this intervention had a short-lived effect on prescribing behavior. On the other hand, since in most cases intervention B started later than intervention A, and some PHCC may have had the meeting at the end of the study period, the available data may not have captured any potential effects of the self-evaluation meeting. We do not either know about the quality of intervention B, i.e. how thoroughly the self-evaluating meeting was performed.

The level of prescriptions at health centers is instead mostly explained by previous prescription levels, showing that it is difficult to change prescription habits. When we exclude all centers that belonged to the quartile with the lowest levels of prescriptions before the interventions started, similar results were obtained. The results are also robust for both antibiotics prescriptions in general and for prescriptions targeted to respiratory tract related infections as well as whether we estimate antibiotics per number of patients visit or per number of listed patients.

We did not find any significant effects for either of the two interventions for private centers. Private healthcare centers were on average smaller, had a different patient composition, and prescribed more antibiotics compared with public centers. This is line with findings by Maun et al. [31]. Our study has several limitations. As we have mentioned, the selection of PHCC was not random. The order of the interventions was not uniform, i.e. while a majority (60%) PHCC underwent intervention A before B, but not all. The quality of the interventions, i.e. how many physicians and nurses that was targeted by intervention A, and how thoroughly the self-evaluating meeting was performed, could not be controlled for. Intervention B was not validated either. Finally, the follow-up time after the interventions varied.

Which other reasons than the two interventions could explain the successful decrease in antibiotic prescriptions? Especially the decrease in inappropriate prescribing, as we showed a larger decrease in prescribing of respiratory tract infection related antibiotics. Moreover, can we find any explanations why the interventions seem not to have played a more significant role? The national Strama organization was established already in the 90-ties in Sweden. During the years before the interventions the discussion of antibiotic resistance and prescriptions of antibiotic among healthcare workers and the public was intensified. The number of prescriptions was also declining already in the years before the interventions took place [32]. This decreasing trend in antibiotic prescriptions was evident in the neighboring Scandinavian countries [32]. Moreover, since a national campaign with the goal of 250 antibiotic prescriptions per 1000 inhabitants was launched in 2011, it is possible that this had started to influence prescription rates before the two interventions occurred at the healthcare center level. The regional Strama organization started informational activities towards both healthcare workers and the public in the first quarter of 2012, with possible further impact of prescription rates.^4^

More important is how many of the permanently employed physicians and nurses and temporary personnel at healthcare centers were targeted by the two interventions, which varied across different centers [33]. It is possible that the interventions were successful at an individual level, among the physician and nurses who participated in the interventions, but it is harder to distinguish the effects of interventions at the healthcare center level. Personnel at the PHCC also believe that structural factors such as the number of staffs, staff turnover, and staff continuity are important reasons behind the prescription levels at their centers [34].

Information on each healthcare center’s rate of antibiotic prescription compared with the other centers was regularly provided by mailings since the second quarter 2013. This information partly replaced intervention A. We conclude that the specific interventions investigated in this study are found to have only modest impact on prescription rates indicating difficulties to find properly operating interventions. Finally, because of the importance of taking account of the dynamics of prescriptions while addressing the potential confounders such as the effects of related initiatives, future studies could benefit from modelling the initial conditions problem and state dependence when analyzing antibiotics prescriptions.

## Conclusions

With this in-depth econometric analysis, we cannot discern a significant, sustainable effect on antibiotic prescribing from either of the two specific interventions, a single educational visit and a self-evaluation meeting. The concurrent successful reduction of inappropriate antibiotic prescribing is due to several factors. Our findings support a multifaceted approach in continuous efforts for prudent use of antibiotics in primary healthcare.

## Data Availability

The data that support the findings of this study are available from Region Västra Götaland but restrictions apply to the availability of these data, which were used under license for the current study, and so are not publicly available. Data are however available from the authors upon reasonable request and with permission of Region Västra Götaland.

## Appendix

**Table 4:** Effects of interventions on total and respiratory tract infection related antibiotics prescription/100 listed patients

	(1)	(2)	(3)	(4)	(1)	(2)	(3)	(4)
VARIABLES	Whole sample	Whole sample	Public centers	Private centers	Whole sample	Whole sample	Public center	Private center
Prescription previous	0.429^***^	0.415^***^	0.0932	0.537^***^	0.516^***^	0.501^***^	0.151^*^	0.564^***^
quarter	(0.0751)	(0.0842)	(0.0701)	(0.0851)	(0.0595)	(0.0691)	(0.0677)	(0.0700)
Intervention A (same	− 0.348^**^	− 0.270	− 0.324^*^	− 0.127	− 0.208^*^	− 0.126	− 0.165	− 0.0200
quarter)	(0.117)	(0.139)	(0.133)	(0.229)	(0.0947)	(0.104)	(0.0955)	(0.168)
Intervention A (after one	−0.339^*^	− 0.181	− 0.499^**^	0.00903	− 0.343^**^	− 0.164	−0.373^***^	0.000343
quarter)	(0.144)	(0.186)	(0.159)	(0.306)	(0.118)	(0.137)	(0.100)	(0.232)
Intervention A (remaining	−0.0379	0.258	−0.229	0.659	−0.129	0.197	−0.233	0.653^*^
quarters)	(0.143)	(0.206)	(0.176)	(0.379)	(0.130)	(0.171)	(0.121)	(0.328)
Intervention B (same	−0.0893	−0.00909	0.0159	−0.295	0.00123	0.0583	0.0250	−0.0961
quarter)	(0.133)	(0.146)	(0.138)	(0.244)	(0.115)	(0.120)	(0.0877)	(0.222)
Intervention B (after one	0.179	0.298	0.250	−0.0957	0.248	0.327	0.235	0.150
quarter)	(0.258)	(0.275)	(0.217)	(0.447)	(0.221)	(0.224)	(0.137)	(0.443)
Intervention B (remaining	0.437	0.587	0.716	−0.287	0.275	0.421	0.519*	−0.115
quarters)	(0.495)	(0.514)	(0.387)	(0.590)	(0.337)	(0.348)	(0.234)	(0.529)
Low care need index		0.283	−0.182	0.544		0.155	−0.224	0.261
		(0.454)	(0.210)	(0.751)		(0.277)	(0.157)	(0.458)
High care need index		0.759^*^	0.604	0.778		0.510*	0.245	0.566
		(0.312)	(0.437)	(0.442)		(0.216)	(0.217)	(0.391)
Adjusted clinical groups		0.615	0.972	−0.0103		−0.984	0.451	−1.258
		(1.508)	(1.269)	(1.986)		(0.977)	(0.801)	(1.360)
Share younger patients		0.506^***^	0.286^***^	0.467^***^		0.334^***^	0.156^***^	0.332^***^
		(0.0827)	(0.0574)	(0.115)		(0.0525)	(0.0364)	(0.0781)
No. of listed patients		−0.377*	−0.465^***^	−0.317		−0.186	− 0.174^**^	−0.186
		(0.151)	(0.0986)	(0.183)		(0.105)	(0.0554)	(0.140)
Constant	3.511^***^	−0.632	4.103	−0.381	1.655^***^	−0.214	1.396	0.180
	(0.407)	(2.521)	(2.110)	(2.494)	(0.197)	(1.599)	(1.189)	(1.816)
Quarterly dummies	Yes	Yes	Yes	Yes	Yes	Yes	Yes	Yes
Observations	1886	1886	1120	766	1886	1886	1120	766
Number of id	189	189	112	77	189	189	112	77

Robust standard errors in parentheses

*** p < 0.001, ** p < 0.01, * p < 0.05

## Abbreviations

ATC: Anatomical Therapeutic Chemical Classification
CNI: Care Need Index
GMM: Generalized Method of Moments estimator
PHCC: Primary HealthCare Center
Strama: The Swedish strategic program against antibiotic resistance

## Footnotes

1 As sensitivity analyses, we also used both types of antibiotics prescriptions (total antibiotics prescriptions and respiratory infection related antibiotic prescriptions) per *number of listed patients* instead of *per patient visits*. See Table A1 in the Appendix.

2 Despite its multiple advantages, this estimate does not allow modelling time-varying unobserved characteristics of clinics that may be correlated with the error term. Because this problem is likely to take place due to the non-randomness of the interventions, the empirical analysis addresses this potential problem in two different ways. First, the inference relies on the finite-sample correction to the reported standard errors in two-step estimation [26], without which those standard errors tend to be severely downward biased [30]. Second, the specification includes a set of time-varying observable characteristics of clinics that take account of the selection criteria used by Strama. The specification also includes a set of quarter dummies, which capture both unobserved time-varying characteristics of clinics and other time-varying trends/events potentially affecting antibiotic prescriptions and infection visits.

3 Because autocorrelation problems can be worsened due to the potential existence of unobserved time-varying characteristics of clinics, making our estimates inconsistent, we also conducted the Arellano-Bond test for zero autocorrelation in first-differenced errors to the estimated models in Table 2. Results evidence that: (1) the Arellano-Bond moment conditions are valid, (2) our estimated models are not miss-specified, and (3) serial autocorrelation is not problematic (i.e., we cannot reject the null hypothesis of no serial correlation in the first-differenced errors at order two). This indicates that these potential problems were addressed in our empirical strategy, suggesting that our estimates are consistent.

4 A possible explanation for the negative trend on the level of prescriptions might be a bundle of other activities by Strama in Region Västra Götaland. This bundle includes educational meetings for all staff at PHCCs, both doctors and nurses, and an organisation with a general practitioner who is locally responsible for ensuring that prescribing is done according to guidelines and coordinating initiatives for prudent antibiotic use.
